# The Foreign Journals: Medical Jurisprudence and Police, Toxicology

**Published:** 1840-01

**Authors:** 


### MEDICAL JURISPRUDENCE, MEDICAL POLICE, TOXICOLOGY.

### *Case of a Person found Hanged, with Marks of Violence on the Head.* By Dr. Heyfelder, of Sigmaringen.

On the 24th July, 1838, the deceased, who was confined in prison, was found dead, hanging in his cell, when only a few hours before he was seen to be in good health and spirits. The key being in possession of the keeper, no one could have had access to the cell. The cell, which was of large size, had one window, secured by an iron grating, about two feet from the floor.

On the sharp projecting part of the window-sill, traces of dried blood mixed with hair, corresponding to that of the deceased, were found: some streaks of dried blood extended from this point to the floor.

The deceased was hanging to one of the door-posts, his feet in contact with the floor, his head inclined on the thorax; both arms rigid and placed over the abdomen, and the fingers curved inwards. He had used a silk handkerchief for the purpose of suspension; and crossing this round the nape of the neck, had lodged the ends between the door and the post. The deceased's mouth was blocked up by his own pocket-handkerchief. He was dressed in the prison dress, and neither on this nor in the cell around were any marks of violence or resistance perceptible. On the forehead and left side of the head were traces of dried blood. He was about thirty-two years old.

The following appearances were found on making a medical inspection of the body:

In the middle of the left parietal bones there were two wounds, with lacerated and swollen edges, surrounded by dried blood. One of these wounds was an inch and a half long; the bones beneath were uninjured. On the right side there were several smaller wounds, all having a lacerated character, and one extending through the cartilage of the right ear. The mark produced by the ligature on the neck was about three fourths of an inch wide, the skin was brown, and here and there corrugated. This mark was situated between the os hyoides and the larynx: there was no ecchymosis in its course. The trachea and its main branches were filled with a white frothy mucous liquid. The lungs and right side of the heart were congested with liquid blood.

The medical opinion given, was, that the deceased had died by hanging, and that he had hanged himself. This opinion was founded on the following facts:

The wounds on the head, which were evidently inflicted during life, were too unimportant in their character to be connected with the death of the deceased. They were superficial, and neither the bones nor the contents of the cranium had suffered from the violence which had produced them.

The state of the trachea, lungs, and heart indicated death from asphyxia, brought on, undoubtedly, by hanging, assisted by the occlusion of the mouth with a handkerchief. The operation of this double cause would have rendered death more rapid than in common cases of hanging. The absence of ecchymosis, from the mark produced by the ligature, might lead some to doubt whether the deceased had really died by hanging: but it is now well ascertained, that an ecchymosed mark is by no means a necessary accompaniment of suspension during life.

It now remains to be explained, how the facts warranted the idea of suicide:

On the supposition of this having been a case of murder, it must be assumed that the deceased was violently seized by his assailants, his head knocked repeatedly against the projecting window-sill: and when thereby stunned, he must have been dragged to the door, strangled with his own cravat, and afterwards hanged. Against this view there were the following circumstances: 1, The prison was so secured, that no one could possibly obtain admittance without the knowledge of the keeper; 2, the absence of all marks of resistance or struggling in the prison or on the person of the deceased, who was in the prime of life, and well able to resist any murderous attack; 3, the tranquil state of the countenance, with the course of the ligature around the neck. Strangulation, while in the erect position, the feet being supported, might seem to militate against suicide, did we not know that there are many cases of suicide on record, in which the deceased have been thus found.

The wounds on the head were doubtless inflicted by his knocking this part of his body against the sharp edge of the window-sill in previous attempts at suicide. Not succeeding in these, he suspended himself with his cravat, having placed the handkerchief in his mouth, in order to accelerate death.

*Henke's Zeitschrift.* 1 H. 1839.

[REMARKS.—We agree with the reporter of this case, that it is well deserving of being made generally known; since, had the body of the deceased been discovered under other circumstances, a well-founded suspicion of murder might have arisen. When a man is found hanged, and circumstances show that he has not died by hanging, this is alone presumptive of murderous interference. How cautious, then, should we be in judging from circumstances! There are, perhaps, even now, professional men who would declare, from the non-production of ecchymosis by the ligature, and the fact of a hanged man being found with his feet in contact with the floor, that such a person could not have died by hanging. An opinion of this kind would not certainly be expressed by one who had attended to the subject, for these questions have long been settled by experience: but there is little doubt that many, without being aware of their error, would not hesitate to give this opinion at inquests. Wounds found on the head, and a handkerchief filling up the mouth of a deceased person, are, however, circumstances which might mislead even an experienced practitioner. The wounds, in the case before us, were, it is true, not of any great extent; but they were in an unusual situation for self-infliction, and did not possess the characters of those commonly found on the persons of suicides. The other fact was still more striking: we do not remember to have ever met with a case of suicide by hanging, in which the deceased stopped up his own mouth so as to prevent respiration. In some future criminal trial, a witness may be asked whether such an act as this is possible in suicidal hanging? This case informs us, that it is not only possible, but has actually been accomplished.]

### *Signs of Death from Hanging.* By M. Orfila.

In our last Number we gave some account of M. Devergie's observations on this subject. M. Orfila has now answered them in the First of a Series of Six Memoirs on subjects of Medical Jurisprudence, which he has presented to the Royal Academy of Medicine. The question he proposes is this: Is it true that we can determine, by the state of the genital organs, whether suspension took place during life or after death? The facts which M. Orfila advances to prove the negative are full of both physiological and legal interest.

You have not forgotten, says M. O., that M. Devergie has read to the Academy a note relative to two signs of suspension during life: viz., the presence of semen in the urethra and the congestion of the genital organs. The observations, on which I deny the sufficiency of these signs, are as follow: 1st. Not only is it possible to find the zoospermata in the urine, which is *first* passed an hour after seminal emission, but even eight, ten, or twelve hours after. Semen, therefore, remains in the urethra of those who have had an emission, till they pass urine, and hence in a case of natural death, or of death by poison, &c., if, for the purpose of deception, the body were hanged after death, and the patient had not passed urine between the time of a seminal emission and death—in any of these cases, the presence of semen in the urethra would be of no value in deciding whether the hanging preceded or was subsequent to death.

2d. I have examined the urethra in the bodies of five persons who had died of different diseases, and had remained lying on their backs, and I have constantly found semen in it. A man, æt. sixty-eight, was examined four hours after death from cancer; the urethra was full of a viscid, lightly amber-coloured liquid, in which there were a great number of seminal animalcules. A man was examined eight hours after assassination; on pressing the urethra, three drops of a milky liquid were obtained, in which a considerable number of animalcules were discerned. In a man of twenty-one, who died of consumption, and was examined thirty hours after; in another, aged forty-six; and in a third, aged sixty-two, examined three hours after death, animalcules were also found in the mucus of the urethra. In five other subjects, only mucus and urine were found in the urethra. The preceding facts sufficiently prove, that the animalcules may be found in the urethra of persons dying of various diseases, and that they are, therefore, of no value as signs of death from hanging.

The next series of examinations relates chiefly to the congestion of the organs of generation. A man, æt. twenty, was examined seven hours after death. The skin of the penis and scrotum was scarcely coloured; the penis, close by the scrotum, was three inches and five lines in circumference. The body was placed upright, and kept in that position for seven hours; the lower extremities were then found very much injected, the penis and scrotum deep violet-coloured, and the former had increased seven lines in circumference. A man, æt. fifty, was hung four hours after death from phthisis, and remained hanging for sixteen hours. Before suspension, the trunk and limbs were still warm and pale; the genital organs were pale, except the prepuce, which had a violet tint; the penis was four inches and a quarter in circumference. After the suspension, the legs were violet to above the knees; the vena dorsalis penis and its principal branches were very evidently distended; there was no erection, but the circumference of the penis was increased four lines. There was a large drop of turbid liquid at the orifice of the urethra, which contained a number of dead seminal animalcules. A man, æt. fifty, was examined three hours after death; the lower extremities were pale, the penis three inches and one line in circumference, and, as well as the scrotum, uncoloured; the orifice of the prepuce was full of a viscid liquid, containing a prodigious number of seminal animalcules. The body was hung, and eight hours after, the scrotum and penis were violet, the circumference of the latter was increased seven lines, and the meatus still contained animalcules. In a fourth case, a man, æt. forty-nine, was hung five hours after death, and remained suspended three hours and a half. The penis, which had before been slightly turgid, was now *erect*, and formed almost a right angle with the abdomen : it had increased nine lines in circumference, it was violet-coloured, and all the veins about it were very much distended. The vesiculæ seminales were very full, and at the orifice of the urethra there was a drop of viscid fluid, containing a great number of animalcules, of which many were alive.

The conclusion which M. Orfila adopts from these facts is, that although it is true that in cases of hanging during life, semen containing some living animalcules is most commonly found in the urethra after death, and the genital organs are so congested, that there may even be erection, yet we should be cautious of concluding, as M. Devergie would, from these circumstances, that the body has been hanged during life; for it is not rare to find semen in the urethra of those who, after dying of various diseases, have remained lying on their backs : and, on the other hand, we may, by hanging bodies even three or four hours after death, and leaving them in that position for some hours, produce a considerable congestion of the genital organs and even erection, and may find animalcules in the urethra, many of which may still be alive. This sign, moreover, is of the less value, since it has been already observed after several other modes of death besides that from hanging.

*Bulletin de l'Académie Royale de Médecine. Sept.* 15, 1839.

---

### *Case of Poisoning by Rotten Eggs.* By Dr. MARCHAL.

DR. MARCHAL was summoned, at eight o'clock one morning, to a carpenter, who was supposed to have had an attack of apoplexy. He learnt that the man had eaten freely on the previous evening, and that since midnight he had been in a state of coma, from which it was impossible to arouse him. He was at this time lying on his back; his face was rather livid, especially about the eyes; his lips were blue; his eyes open and fixed; there was no distortion of the mouth; the limbs were flaccid; the pulse small and regular; the respiration slow. His wife, his brother, and one of his sons were affected, though in a less degree, with the same symptoms, complaining of vertigo, weight and pain in the head, pains in the limbs, and disinclination to move. When questioned on the suspicion of their having taken some narcotic poison, the patients stated that on the previous evening they had all eaten of a pudding, made with frozen eggs, (the thermometer was, at the time, 8° below zero, Centigrade,) and to these they attributed all their symptoms. They added, that the internal surface of the shells was greenish, and that the eggs had a disagreeable slightly putrid smell. It was only a short time after their meal that they all felt indisposed. The man himself was largely bled from the arm, but without relief. Leeches and ice were applied to his head, and large mustard poultices to each calf; but it was not till after three hours that he began to arouse from his state of narcotism, and to recognize those around him. He had afterwards no recollection of what happened; he only remembered that at the beginning of his sleep he was tormented with frightful dreams. He recovered completely, but rather slowly; and a slight paralysis of the left little finger remained for a considerable time.

On the evidence of four persons being similarly affected, after eating eggs in which putrefaction had commenced, though it was probably retarded by their being frozen, and the resemblance of their symptoms to those which are known, from the observations of Chaussier and others, to result from the application of sulphuretted hydrogen gas, there seems little reason to doubt that the gas generated in the decomposition of the eggs was the common cause of the simultaneous seizure of the patients. [We beg leave to question this explanation.]

*Gazette Médicale. Juin* 22, 1839.

*Suspected Poisoning:—a warning case.* By Dr. STEINBECK, of Brandenburg.

SEVERAL years ago, says Dr. S., a case occurred to me of great interest in a pathological, but especially in a medico-legal point of view; for if a little less accurate examination of the body had been made, a judicial murder might have been very readily committed. A young and strong ploughboy was taken, while out washing sheep, with the most frightful pain in the region of the stomach, and died in *eight hours* with symptoms which led to the suspicion of poisoning. This was the more readily entertained, as the young man had two sweethearts, and had long wavered in his choice. She who was slighted was known to be a very violent and revengeful girl, and it so happened that this very person gave the patient some drink shortly before he was taken ill. The chemical analysis of what the patient vomited and of the contents of the stomach and intestines showed, however, no trace of poison. But there was found in the stomach a hole, the size of a shilling; and it appeared that the stomach had been adherent to the spleen, and that by forcible separation of this adhesion, the hole in the stomach was caused, and death thereby brought on. It was now told that the deceased had challenged another to box, that he had again refreshed himself from the young woman's pitcher, that then he fought with everybody, and was thrown to the ground, whereupon he cried out. After this, he got up without saying a word, pale (with anger, according to the bystanders), and went to his work. On account of the raging thirst which now set in, he drank several times from the said pitcher,—a circumstance which he himself gave as the cause of his illness to the judicial authorities shortly before his death. *Casper's Wochenschrift. May* 18, 1839.

---

*On the Poisonous Effects of Copaiba.* By M. SANDRAS.

A MAN, æt. 25, contracted a gonorrhœa, for which he was treated by an advertising quack. In a few weeks the discharge ceased. He entered La Pitié on the 13th August, for a pulmonary affection, which was soon cured; but his urethral discharge returned. The *potion de Chopart,* which contains copaiba, was prescribed for him; but it was discontinued, after two days' use, and an enema was ordered, containing half an ounce of the balsam. This was soon followed by pain at the stomach, vomiting, and general uneasiness; on the following day epileptic convulsions occurred, to an alarming extent; for three days he was unable to speak; it was necessary to tie him in bed, to prevent his sustaining injury during the convulsion. Cupping-glasses and moxas were applied by the side of the cervical vertebra; ice to the head; sinapisms to the feet and hands; and calomel was administered freely. Under the use of these means the convulsions ceased, and the nervous symptoms subsided. Twenty-five days after the attack he was detained in the hospital merely by the suppuration of the moxas. He went out perfectly well at the end of two months.

In this case no other cause could be assigned for the invasion of the symptoms than the administration of the copaiba. We must, therefore, place it among those instances of peculiar idiosyncrasy in which we find substances, frequently the most innocuous, become poisonous to particular individuals.

*Bulletin général de Thérapeutique. Feb.* 1837.

